# Multiplex PCR Unyvero i60 ITI application improves detection of low-virulent microorganisms in periprosthetic joint infections

**DOI:** 10.1007/s00264-018-4136-z

**Published:** 2018-09-06

**Authors:** Irene Katharina Sigmund, Reinhard Windhager, Florian Sevelda, Kevin Staats, Stephan Engelbert Puchner, Sandra Stenicka, Florian Thalhammer, Johannes Holinka

**Affiliations:** 1 Department of Orthopaedics and Trauma Surgery, Medical University of Vienna, Spitalgasse 23, 1090 Vienna, Austria; 2 Department of Internal Medicine I, Division of Infectious Diseases and Tropical Medicine, Medical University of Vienna, Spitalgasse 23, 1090 Vienna, Austria

**Keywords:** Multiplex PCR system, Periprosthetic joint infection, Low-grade infections, Low-virulent microorganisms, Diagnosis

## Abstract

**Purpose:**

The aim of this study was to evaluate the pre-operative performance of an automated multiplex PCR (mPCR) system in patients with suspected periprosthetic joint infection (PJI).

**Methods:**

Under sterile conditions, synovial fluid samples from patients with a suspected PJI were collected pre-operatively. One hundred eighty microliter of the aspirate was used for analysis in the mPCR. The remaining joint fluid was sent for microbiological analysis. PJI was diagnosed by using the Musculoskeletal Infection Society (MSIS) criteria. Total percentage agreement and Cohen’s kappa coefficient were calculated to measure overall agreement.

**Results:**

Overall, 90 patients with a suspected PJI were included. Using MSIS criteria, 38 (42%) patients were classified as septic. Total percent agreement between mPCR and synovial fluid culture was 86% with a Cohen’s kappa of 0.68. The mPCR and synovial fluid culture showed sensitivities of 71% and 84%, respectively. Combined evaluation provided an even higher sensitivity of 92%. While *Cutibacterium* spp. were detected five times by mPCR, it could only be cultured once. A higher detection rate of CoNS by mPCR (*n* = 7) compared to conventional culture (*n* = 5) was also demonstrated. In comparison to synovial fluid culture, the mPCR missed *Staphylococcus aureus* five times.

**Conclusion:**

With a moderate agreement between synovial fluid mPCR and culture, the mPCR system could be a useful adjunct in diagnosing a PJI pre-operatively. Due to faster availability of results and a higher detection rate of low-virulent microorganisms, it can complement conventional culture.

## Introduction

The pre-operative detection of the causative microorganism(s) and antimicrobial susceptibility is essential for ideal surgical and antibiotic treatment of periprosthetic joint infection (PJI). An accurate and prompt evaluation is crucial for treatment success. Currently, pre-operative-collected synovial fluid cultures represent the method of choice for diagnosing an infection and planning the optimal revision surgery (one-stage vs. two-stage). Results from conventional microbiological culture-based analyses should become available within one to 14 days [1]. If a PJI with low-virulence microorganisms is suspected, the cultures should be observed for 14 days or longer [1]. Treatment is adjusted according to the antibiogram. This time period in which treatment is considered is needed to choose the ideal operation procedure and to optimize antimicrobial therapy.

Currently, attention has been paid to polymerase chain reactions (PCR) for genotypic evaluation of bacteria. These techniques are verified methods in diagnosing infectious diseases in hospitalized patients. In the literature, multiplex PCR analysis of periprosthetic tissue or periprosthetic sonicate fluid samples has already been evaluated for diagnosing orthopaedic infections [2–5]. The great advantages of multiplex PCR systems are the short turnaround time and rapid identification within five to six hours of the most common clinically relevant pathogens and genetic markers of resistances.

The purpose of the current study was to investigate the pre-operative performance of the automated multiplex PCR Unyvero i60 ITI cartridge application (Curetis GmbH, Holzgerlingen, Germany) in patients with suspected periprosthetic joint infection under clinical conditions. We aimed to evaluate the detected pathogens and resistance marker in synovial fluid samples so as to compare the results with those of synovial fluid cultures. Furthermore, the merit of combining both diagnostic methods was determined.

## Material and methods

### Study design

This prospective cohort study was conducted in a tertiary healthcare centre. Synovial joint fluid samples were investigated by a specific commercial mPCR system and conventional culture. The study was approved by the institutional ethical review board of Medical University of Vienna and was done in accordance with Declaration of Helsinki.

### Study population

Between March 2016 and February 2018, 98 consecutive patients older than 18 years with a painful prosthesis and suspected periprosthetic joint infection were included. Exclusion criteria were invalid test results of the multiplex PCR system, obvious contamination of the joint fluid as well as contamination during transport to the laboratory, and an incomplete dataset. A periprosthetic joint infection was defined according to the Musculoskeletal Infection Society (MSIS) criteria [1].

### Multiplex PCR evaluation

In the context of routine clinical pre-operative diagnosis of a painful periprosthetic joint, a needle aspiration of the affected joint was performed under sterile conditions in the outpatient facility before revision surgery. One hundred eighty microliter of the collected synovial fluid was used for microbial analysis using the multiplex PCR Unyvero i60 ITI application (Curetis GmbH, Holzgerlingen, Germany), which covers more than 100 pathogens and resistance markers. The remaining joint fluid was sent for conventional microbiological analysis.

The Unyvero i60 ITI application is a semiquantitative DNA test capable of parallelly performing eight multiplex PCR reactions to detect the nucleic acid of specific pathogens commonly found in PJI. It further can provide information about antibiotic resistance genes.

All specimens were processed with the Unyvero i60 ITI application according to the manufacturer’s protocol as previously described [6]. All performed tests were purchased by our department. A sample was seen to be positive if at least one of the analytes (pathogens) reached the threshold of 10^4^ DNA fragments/pathogen/ml. The detection threshold for the antibiotic resistance analytes was determined with serial dilutions of living bacteria or dilutions of DNA fragments in the buffer by the manufacturer. The resistance markers *aacA4*, *ctx*-*M*, *ermA*, *mecA*, *ndm*, *oxa*-*23*, *oxa*-*48*, *oxa*-*58*, and *vanA* were detected at a concentration of 10^4^ DNA fragments/pathogens/ml; *aac*(*6′*)/*aph*(*2″*), *gyrA*, *imp*, *kpc*, *oxa*-*24*, and *vim* were identified at a concentration of 10^5^ DNA fragments/pathogens/ml; and *ermC*, *mecC*, *rpoB*, and *vanB* were detected at a concentration of 10^6^ DNA fragments/pathogens/ml.

### Routine cultivation of synovial fluid

Synovial fluid (SF) was inoculated onto the following media: Columbia agar III with 5% sheep blood and chocolate agar with IsoVitaleX and bacitracin (BD, Heidelberg, Germany). Both media were incubated in a carbon dioxide-enriched atmosphere for up to 14 days. McConkey agar No. 3 (Oxoid, Basingstoke, UK) was incubated for 48 hours at aerobic conditions. Brucella Blood agar with Hemin and vitamin K1 and Schaedler kanamycin-vancomycin with 5% sheep blood (BD, Heidelberg, Germany) were incubated under anaerobic conditions for up to 14 days. Additionally, an in-house-prepared brain heart infusion with 0.1% agar was also inoculated and incubated for up to 14 days. All media were incubated at 35–37 °C. Susceptibility testing was performed using the EUCAST disc diffusion method and interpreted according to the EUCAST clinical breakpoints for bacteria v 5.0.

### Diagnostic tests

Serum CRP-levels were determined pre-operatively. In line with proceedings of the International Consensus Meeting [1], a cutoff of 10 mg/l was maintained. If the cutoff was exceeded, the minor criterion “serum CRP-level” was classified as positive. To evaluate the leukocyte count, a minimum of 1 ml synovial fluid was analyzed in an automatic manner. During surgery (if performed), at least three periprosthetic tissue samples were sent for microbiological analysis as mentioned above.

## Statistical analysis

Positive and negative percent agreement between conventional culture and the multiplex PCR system was calculated as the number of concordant positive (negative) observations divided by the number of positive (negative) results of conventional culture. Total percentage agreement and Cohen’s kappa coefficient were calculated to measure overall agreement. Sensitivity, specificity, positive (LR+) and negative likelihood ratio (LR−), area under the ROC curve (AUC), positive (PPV) and negative predictive value (NPV) of mPCR, conventional culture, and the combination of both diagnostic methods were calculated, and individual receiver operating characteristic (ROC) curves were drawn for each test. All estimated parameters are reported with 95% confidence intervals. The software XLSTATPM (version 2017; XLSTAT; Addinsoft, New York, NY, USA) was used to perform the statistical analysis.

## Results

### Demographics and infection classification

Eight patients had to be excluded because of incomplete data. Ninety patients (47 female, 43 male) with a suspected periprosthetic joint infection were eligible for inclusion. The median age was 72 (range, 25–88) years. Fifty-one (57%) patients had a suspected infection after a total knee arthroplasty (TKA), 33 (37%) after a total hip arthroplasty (THA), four (4%) after a total shoulder prosthesis, one (1%) after a total upper ankle joint prosthesis, and one (1%) after a total elbow prosthesis. Twenty-six (29%) patients received antibiotics before aspiration.

Thirty-eight patients (42%) out of the 90 cases with suspected PJI were classified as septic according to the MSIS criteria. Of these 38 patients, at least one microorganism was detected in 27 synovial fluid samples by mPCR and in 32 synovial fluid specimens by SF culture (Fisher’s exact test; *p* = 0.271).

### Performance of the multiplex PCR system

Overall, 34 microorganisms out of 29 synovial fluid samples could be detected in the mPCR system. In the conventional culture, 34 microorganisms out of 32 synovial fluid sample cases showed microbial growth. Overall percent agreement between mPCR and SF culture was 85.6% (95% CI, 78.3–92.8%). A Cohen’s kappa of 0.68 (0.52–0.84) indicated moderate agreement between mPCR and conventional culture of the SF. We calculated the positive and negative percent agreement in all cases. Furthermore, we separated the cohort in subgroups and calculated the overall percent agreement in patients after a THA and TKA, as shown in Table 1. A slightly higher overall agreement in patients after a TKA could be demonstrated, but it was not statistically significant (chi-squared test; *p* = 0.99).

**Table 1:** Positive, negative, and overall percent (%) agreement and Cohen’s kappa coefficient between the mPCR system and the conventional culture of synovial fluid samples in the total study cohort, after total knee arthroplasty (TKA) and total hip arthroplasty (THA) with their 95% confidence interval

	Positive % agreement	Negative % agreement	Overall % agreement	Kappa
Total	75.0 (57.6–86.9)	91.4 (80.8–96.6)	85.6 (78.3–92.8)	0.68 (0.52–0.84)
TKA	73.7 (50.8–99.4)	93.8 (78.6–99.2)	86.3 (76.8–95.7)	0.70 (0.49–0.91)
THA	72.7 (42.8–90.5)	86.4 (65.6–95.9)	81.8 (68.7–95.0)	0.59 (0.29–0.89)

Table 2 shows the distribution of all detected microorganisms according to the mPCR and conventional culture of the collected synovial fluid. The multiplex PCR system identified *Staphylococcus aureus* (*n* = 13) and coagulase-negative Staphylococci (*n* = 8) as the most common pathogens. Regarding conventional culture, the most frequently found bacterium was *Staphylococcus aureus* (*n* = 18) followed by coagulase-negative Staphylococci (*n* = 5). Particularly noteworthy are the differences of the identified microorganisms in the mPCR and the conventional culture. *Cutibacterium* spp. were detected in five specimens by the multiplex PCR system, whereas the conventional culture was only able to isolate *Cutibacterium avidum*/*granulosum* in one sample. A higher detection rate of coagulase-negative Staphylococci by mPCR (*n* = 7) compared to the conventional culture (*n* = 5) was also demonstrated. On the other hand, the conventional culture was able to isolate *Staphylococcus aureus* in 18 specimens; the mPCR system identified this pathogen in 13 synovial fluid samples. No statistically significant difference was shown between the performance of the mPCR in patients with and without antibiotics before aspiration (Fisher’s exact test; *p* = 0.457).

**Table 2:** Distribution of microorganisms detected in the synovial fluid (SF) by multiplex PCR (mPC) and conventional culture in the study cohort

Isolated microorganism	mPCR (*n* = 29)	SF culture (*n* = 32)
*Staphylococcus aureus*	13	18
Coagulase-negative Staphylococci	7	5
*Cutibacterium avidum*/*granulosum*	2	1
*Cutibacterium acnes*	3	0
*Pseudomonas aeruginosa*	2	2
*Streptococcus* spp.	2	2
*Escherichia coli*	1	1
*Enterococcus faecalis*	1	1
*Klebsiella pneumoniae*	1	0
*Proteus* spp.	1	1
*Finegoldia magna*	1	1
*Enterobacter cloacae* complex	0	1
*Actinomyces neuii*	0	1
Total	34	34

**Fig. 1:**
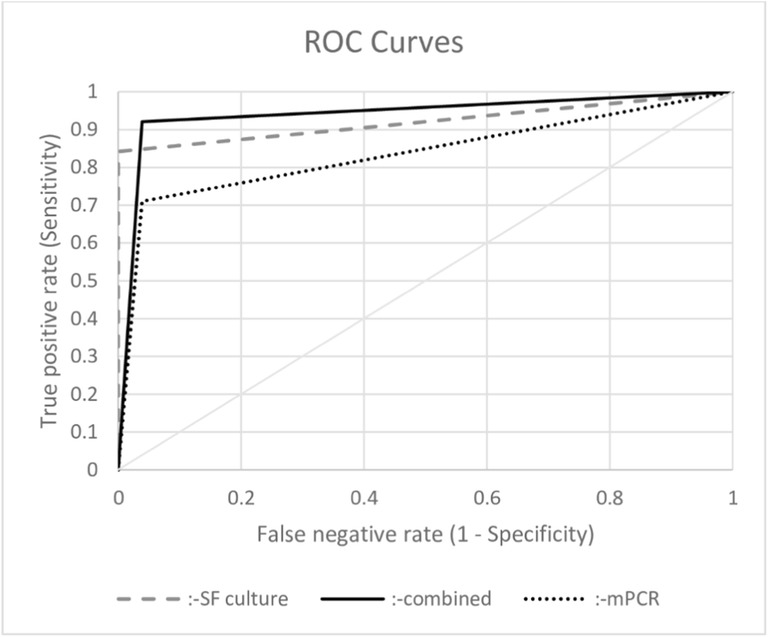
Receiver operating characteristic curves for diagnostic accuracy of periprosthetic joint infection based on multiplex PCR (mPCR), synovial fluid culture (SF culture), and the combination of both test methods (combined)

The sensitivity and specificity of the mPCR were 71.1% (95% CI, 55.1–83.0) and 96.2% (95% CI, 86.1–99.6), respectively. A sensitivity and specificity of synovial fluid cultures were calculated with 84.2% (95% CI, 69.1–92.8) and 100.0% (95% CI, 91.6–100), respectively. The combined evaluation of mPCR and synovial fluid cultures showed a sensitivity of 92.1% (95% CI, 78.3–97.9) and a specificity of 96.2% (95% CI, 86.1–99.6), respectively. Sensitivity, specificity, LR+, LR−, AUC, PNV, and NPV of the mPCR, conventional culture, and combined evaluation of both test methods are shown in Table 3, and their ROC curves are drawn in Fig. 1.

**Table 3:** Sensitivity, specificity, positive and negative likelihood ratio (LR+, LR−), area under the curve (AUC), and positive (PPV) and negative predictive value (NPV) with their 95% confidence interval of the multiplex PCR system, conventional culture of the synovial fluid (SF) samples, and the combination of both test methods according to the MSIS criteria

	mPCR	Conventional culture SF	Combination
Sensitivity	71.1% (55.1–83.0)	84.2% (69.1–92.8)	92.1% (78.3–97.9)
Specificity	96.2% (86.1–99.6)	100.0% (91.6–100)	96.2% (86.1–99.6)
LR+	18.47 (4.68–72.99)	–	23.95 (6.13–93.51)
LR−	0.30 (0.18–0.50)	0.16 (0.08–0.33)	0.08 (0.03–0.24)
AUC	0.84 (0.76–0.91)	0.92 (0.86–0.98)	0.94 (0.89–0.99)
PPV	93.1% (83.9–100)	100% (100)	94.6% (87.3–100)
NPV	82.0% (72.3–91.6)	89.7% (81.8–97.5)	94.3% (88.1–100)

In five patients, the mPCR system was able to identify pathogens, while the synovial fluid culture was not able to do so (*Cutibacterium acnes* [*n* = 1], coagulase-negative Staphylococci [*n* = 1], *Cutibacterium acnes* + coagulase-negative Staphylococci [*n* = 1; both in one patient], coagulase-negative Staphylococci [*n* = 1]). Out of these, two were classified aseptic, although definitive histology indicated a low-grade infection (according to Krenn classification type III), and the serum CRP-level was elevated. It is particularly noteworthy that these patients were under antimicrobial treatment. In other three mPCR-positive patients, the MSIS criteria were positive, but no microorganism was isolated in the synovial fluid culture.

Although an infection was present (according to the MSIS criteria) in eight cases, no microorganism was identified by mPCR, while the synovial fluid culture was positive (*Staphylococcus aureus* [*n* = 4], coagulase-negative Staphylococci [*n* = 2], *Enterobacter cloacae* complex [*n* = 1], *Actinomyces neuii* [*n* = 1]).

Three synovial fluid samples showed negative results in mPCR and SF culture, although an infection was diagnosed (*Staphylococcus aureus* [*n* = 1; tissue culture]; coagulase-negative Staphylococci [*n* = 1; tissue culture, sonication]; negative culture [*n* = 1]). In one synovial fluid sample with suspected PJI, the detected pathogens did not match. The multiplex PCR system detected *Klebsiella pneumoniae*, and in the conventional culture of the synovial fluid, *Staphylococcus aureus* was isolated. *Staphylococcus aureus* was also cultured in the tissue and sonication fluid, and in the blood cultures, *Klebsiella pneumoniae* was isolated.

In two synovial fluid samples, *Cutibacterium acnes* was detected by the mPCR in addition to *Staphylococcus aureus* (in one patient) and *Cutibacterium avidum* (in the other patient). However, the conventional culture only isolated *Staphylococcus aureus* (in the first patient) and *Cutibacterium avidum* (in the second patient).

### Performance of resistance detection

Table 4 shows the resistance marker detected by the mPCR system in comparison with the conventional culture of all corresponding microorganisms. Nine concordant microorganisms were sensitive and did not show a resistance either in the conventional culture or in the mPCR system. Overall, the mPCR system found 11 gene resistance markers, and the conventional culture of the synovial fluid samples found 23 antibiotic resistances. In one *Cutibacterium avidum*/*granulosum*, a resistance to clindamycin was detected by conventional resistance analysis. Due to a lack of resistance gene markers, the mPCR was not able to identify this resistance. Nevertheless, in this bacterium, only the resistance gene marker [*erm X*] is described in the literature [9]. Therefore, the mPCR system could not detect this resistance.

**Table 4:** Detected gene resistance marker in the multiplex PCR Unyvero i60 ITI application compared to the antibiogram of the conventional culture in synovial fluid samples with concordant microorganisms

Concordant microorganisms	mPCR gene resistance marker	Culture-resistance
*Enterococcus faecalis*	Macrolides/lincosamides [*ermC*]	No resistance
Coagulase-negative Staphylococci	Aminoglycosides *aac*(*6*′)/*aph*(*2*″)	Gentamicin
	Macrolides/lincosamides [*ermA*]	Oxacillin
		Ciprofloxacin
*Staphylococcus aureus*	No resistance	No resistance
Coagulase-negative Staphylococci	No resistance	No resistance
*Cutibacterium avidum*/*granulosum*	No resistance	Clindamycin
*Escherichia coli*	Fluoroquinolones [*gyrA*]	No resistance
*Pseudomonas aeruginosa*	No resistance	No resistance
*Staphylococcus aureus*	No resistance	No resistance
*Staphylococcus aureus*	Macrolides/lincosamides [*ermA*]	Erythromycin, clindamycin
	Glycopeptide [*vanB*]	
*Staphylococcus aureus*	No resistance	No resistance
*Staphylococcus aureus*	Macrolides/lincosamides [*ermA*]	Erythromycin, clindamycin
*Staphylococcus aureus*	Rifampicin [*rpoB*]	Fosfomycin
*Staphylococcus aureus*	Macrolides/lincosamides [*ermC*]	Erythromycin, clindamycin
*Pseudomonas aeruginosa*	No resistance	Ciprofloxacin
*Staphylococcus aureus*	No resistance	Erythromycin, clindamycin
*Staphylococcus aureus*	No resistance	Erythromycin, clindamycin
*Proteus* spp.	No resistance	No resistance
*Staphylococcus aureus*	No resistance	No resistance
*Staphylococcus aureus*	No resistance	No resistance
*Staphylococcus aureus*	Macrolides/lincosamides [*ermC*]	Erythromycin, clindamycin
	Aminoglycosides *aac*(*6*′)/*aph*(*2*″)	
*Staphylococcus aureus*	No resistance	Erythromycin, clindamycin
*Streptococcus* spp.	No resistance	No resistance
*Streptococcus* spp.	No resistance	Erythromycin, clindamycin
		Doxycyclin

In one concordant *Enterococcus faecalis*, the drug resistance marker macrolides/lincosamides [*ermC*] was detected only by mPCR. However, macrolides or lincosamides are not clinically relevant.

In one *Pseudomonas aeruginosa*, a resistance to ciprofloxacin was detected by conventional resistance analysis. Due to a lack of resistance gene markers, the mPCR was not able to identify this resistance.

After a mean time period of 2.58 days (SD, 3.87), the microorganism was assessed by conventional culture in patients with concordant microorganisms. The antibiogram of the conventional culture was available for the physician after an average of 3.83 (SD, 4.09) days. While *Staphylococcus aureus* was identified after one day, *Cutibacterium avidum* needed 14 days to be isolated.

## Discussion

The pre-operative diagnosis of PJI remains challenging. While serum CRP and ESR may not be accurate as diagnostic tools in the pre-operative diagnosis of PJI (and identification of infection persistence), particularly to identify low-grade PJI [10, 11], the synovial fluid white blood cell count/differential showed promising results, but cannot identify the causative microorganism [12]. The conventional culture of synovial fluid samples is the current gold standard [13] but has inferior sensitivity in comparison to tissue samples and sonication fluid, and a relatively long time period is needed before results are available [14]. In recent years, attention has been paid to new diagnostic techniques, such as the alpha defensin lateral flow test [12, 15–17] and multiplex PCR techniques [2, 5, 6, 8]. While both test methods are able to confirm PJI, the multiplex PCR system additionally provides information about the causative pathogen.

In the present study, an 86% agreement of all cases between the mPCR system and the conventional culture could be illustrated. This was in line with the results (82%) reported by Morgenstern et al. [7]. According to the MSIS criteria, the sensitivity of the mPCR was 71%. However, it is well known that some infections, especially low-grade infections, might be present without meeting these criteria [18, 19], but we utilized them in our study since they were used in other papers that analyzed diagnostic methods of PJI [19–24]. In contrast to the MSIS criteria, proposed EBJIS criteria are assumed for better detection of low-grade PJI but show the risk to misdiagnose aseptic cases as PJI [12]. Nevertheless, the sensitivity of the mPCR was 52% if we used the EBJIS criteria which is comparable to the reported 60% by Morgenstern et al. [7]. The multiplex PCR system showed superior detection of low-virulent organisms [7]. This phenomenon was also observed in our study. In contrast, this system showed a disadvantage in identifying *Staphylococcus aureus* when it came to diagnosing a PJI, possibly because of the prevailing high threshold value. However, Portillo et al. demonstrated better detectability of *Staphylococcus aureus* [5] by mPCR in sonication fluid samples. To investigate this detail more precisely, further studies with a higher sample size are needed.

A detailed list of results reported in literature is shown in Table 5. Due to different sample material, a proper comparison to the other studies is not possible. However, the ascertained literature data evaluated materials (sonication fluid, tissue samples) removed during surgery when the revision procedure was already planned [2–5]. Most likely, the results of the mPCR or the conventional culture were only available after the surgery was finished. A detailed planning of the operation (one-stage vs. two-stage) should be done pre-operatively. The faster availability of results (5–6 h) and better detection rate of low-virulent organisms of mPCR can provide an earlier decision by the surgeon and therefore an earlier treatment for the patient. Nevertheless, the combined use of synovial fluid culture and mPCR seems to be a better diagnostic tool (sensitivity 92%) than one method alone. Therefore, the SF culture cannot be replaced by the mPCR system and should remain the standard in the preoperative investigation for the diagnosis of PJI.

**Table 5:** Results of the literature with listed test material of each study, overall percent (%) agreement between mPCR and culture, sensitivity and specificity of the mPCR, synovial fluid, sonication, and tissue culture

	Present study	Morgenstern et al. [7]	Renz et al. [6]	Achermann et al. [2]	Portillo et al. [5]	Hischebeth et al. [4]	Borde et al. [3]	Suda et al. [8]
Test material								
mPCR	Synovial fluid	Synovial fluid	Sonication fluid	Sonication fluid	Sonication fluid	“Pooled PCR”	Tissue samples	Tissue samples
Culture	Synovial fluid	Synovial fluid	Sonication fluid	Sonication fluid	sonication + tissue	Sonication fluid	Tissue samples	Tissue samples
Used criteria	MSIS/EBJIS	EBJIS	EBJIS	Modified EBJIS	Modified EBJIS	MSIS	nm	MSIS
Overall % agreement	86%	82%	64%	57%	nm	nm	82%	nm
Sensitivity mPCR	71%/52%	60%	51%	78%	96%	67%	nm	31%
Sensitivity culture								
Synovial	84%/57%	52%	nm	nm	nm	nm	nm	nm
Sonication	86%/66%	61%	58%	62%	71%	89%	nm	nm
Tissue	74%/60%	45%	51%	nm	67%	67%	nm	85%
Specificity mPCR	90%/100%	89%	94%	nm	100%	100%	nm	100%
Specificity culture								
Synovial	100%/100%	98%	nm	nm	nm	nm	nm	nm
Sonication	100%/100%	91%	100%	nm	100%	62%	nm	nm
Tissue	95%/100%	96%	100%	nm	98%	82%	nm	100%

*nm*, not mentioned in the published paper; “*Pooled PCR*”, aspirate and sonication fluid

However, the currently available criteria [25] only include microorganisms isolated by culture and did not mention the detection of a pathogen by another source, such as PCR. This could represent an as-yet undetected problem with these criteria. An infection could be undiagnosed and insufficiently treated. Therefore, the lack of undetected low-virulent organisms could be minimized by additional inclusion of PCR systems.

In 14 concordant microorganisms, resistance or a resistance marker was determined either in the multiplex PCR system or in the SF culture. A positive and negative conformity was illustrated five and eight times, respectively. However, six of the resistance markers identified by conventional culture (oxacillin, clindamycin, erythromycin) were not detected in the mPCR system, even though the resistance marker genes ([*mecA*, *mecC*], [*ermA*, *ermC*]) are included in this device. A limitation of detectable resistance markers by the multiplex PCR system caused a lack in identification of four antibiotic resistances detected by conventional culture (ciprofloxacin [*n* = 2], fosfomycin [*n* = 1], doxycyclin [*n* = 1]). In two concordant microorganisms, only mPCR identified clinical relevant resistance gene markers (*Escherichia coli* fluoroquinolones [*gyrA*], *Staphylococcus aureus* [aminoglycosides *aac*(*6*′)/*aph*(*2*″)]), while the synovial fluid culture was not able to do so. However, due to the small number of detected resistance markers, a recommendation is not possible.

A limitation of the mPCR system is the lack of detectable microorganisms. However, in our study, only one of these undetectable pathogens in the mPCR was isolated in the conventional culture. Hence, it seems that the most common PJI-related microorganisms can be detected by this novel mPCR device.

## Conclusion

The mPCR system provides an advantage in identification of low-virulent microorganisms and has short turnaround time. In comparison with conventional culture, mPCR was inferior for detection of *Staphylococcus aureus*. Nevertheless, with moderate agreement compared to the conventional culture and high sensitivity of combined evaluation, this device could be a useful adjunct in pre-operative diagnosis of PJI, especially in low-grade infections.
